# UPMaBoSS: A Novel Framework for Dynamic Cell Population Modeling

**DOI:** 10.3389/fmolb.2022.800152

**Published:** 2022-03-02

**Authors:** Gautier Stoll, Aurélien Naldi, Vincent Noël, Eric Viara, Emmanuel Barillot, Guido Kroemer, Denis Thieffry, Laurence Calzone

**Affiliations:** ^1^ Equipe Labellisée Par La Ligue Contre Le Cancer, Université de Paris, Sorbonne Université, INSERM UMR1138, Centre de Recherche des Cordeliers, Paris, France; ^2^ Metabolomics and Cell Biology Platforms, Gustave Roussy Cancer Campus, Université Paris Saclay, Villejuif, France; ^3^ Institut de Biologie de L’ENS (IBENS), Ecole Normale Supérieure, CNRS, INSERM, Université PSL, Paris, France; ^4^ Lifeware Group, Inria Saclay-Ile de France, Palaiseau, France; ^5^ Institut Curie, PSL Research University, Paris, France; ^6^ INSERM U900, Paris, France; ^7^ MINES ParisTech, CBIO-Centre for Computational Biology, PSL Research University, Paris, France; ^8^ Sysra, Yerres, France; ^9^ Pôle de Biologie, Hôpital européen Georges Pompidou, AP-HP, Paris, France

**Keywords:** stochastic simulation, cell interactions, heterogeneous cell population, logical model, pathway modeling

## Abstract

Mathematical modeling aims at understanding the effects of biological perturbations, suggesting ways to intervene and to reestablish proper cell functioning in diseases such as cancer or in autoimmune disorders. This is a difficult task for obvious reasons: the level of details needed to describe the intra-cellular processes involved, the numerous interactions between cells and cell types, and the complex dynamical properties of such populations where cells die, divide and interact constantly, to cite a few. Another important difficulty comes from the spatial distribution of these cells, their diffusion and motility. All of these aspects cannot be easily resolved in a unique mathematical model or with a unique formalism. To cope with some of these issues, we introduce here a novel framework, UPMaBoSS (for Update Population MaBoSS), dedicated to modeling dynamic populations of interacting cells. We rely on the preexisting tool MaBoSS, which enables probabilistic simulations of cellular networks. A novel software layer is added to account for cell interactions and population dynamics, but without considering the spatial dimension. This modeling approach can be seen as an intermediate step towards more complex spatial descriptions. We illustrate our methodology by means of a case study dealing with TNF-induced cell death. Interestingly, the simulation of cell population dynamics with UPMaBoSS reveals a mechanism of resistance triggered by TNF treatment. Relatively easy to encode, UPMaBoSS simulations require only moderate computational power and execution time. To ease the reproduction of simulations, we provide several Jupyter notebooks that can be accessed within the CoLoMoTo Docker image, which contains all software and models used for this study.

## 1 Introduction

One of the key challenges in cell biology or in biochemistry is to understand how perturbations of genes, proteins or metabolites affect cellular behavior. For that, the construction and analysis of mathematical models constitute a powerful approach, which can help formalize and reason on the complex phenomena governing the functioning of the cell. At the cellular level, the relations between single entities can be described as signaling, biochemical, or metabolic pathways, and transcribed into mathematical terms to predict the impact of specific perturbations on cellular processes. The difficulty grows when considering inter-cellular signals and their effect at the organ level or the role of the micro-environment on the cell fate. Ideally, the mathematical model should include not only detailed pathway descriptions for every cell, but also key events occurring at the population level, e.g., extra-cellular diffusion, cell motility, inter-cellular communications, death or division of cells.

Multi-cellular systems models have already been studied in developmental biology (Cartwright et al., 2009). Most often, cell populations are considered as a large set of single entities (cells), diffusing and moving throughout the environment, giving rise to reproducible spatial organizations. Formal frameworks borrowed from physics are often used, such as partial differential equations. These approaches allow detailed and accurate temporal and spatial descriptions of collective cellular behaviors (Cowan et al., 2012).

In the field of cancer and immunology, many published models considered a generic cell (e.g., an epithelial cell, a T lymphocyte cell, a macrophage, etc.) to represent the behavior of cell populations. An extension of this simplistic view relies on stochastic simulations to estimate the evolution of desynchronized cell populations. Although such studies do not consider explicitly individual cells (Shmulevich et al., 2002; Albert et al., 2008; Stoll et al., 2017), the outputs of these simulations can be interpreted as the composition of a population of non-interacting cells.

In both cancer (Anderson et al., 2006) or auto-immune diseases (El-Badri et al., 2007), there are different cell types that need to be considered. In this respect, agent-based approaches associate an agent with each cell, which activity depends on that of its neighbors (Bonabeau, 2002; Altinok et al., 2011). Powerful tools have been developed to define and analyze such models, including CellSys (Drasdo and Hoehme, 2010), CompuCell3D (Swat et al., 2012) or PhysiCell (Ghaffarizadeh et al., 2018).

However, these models usually do not explicitly take into account intra-cellular signaling pathways, or the specific deregulations that may occur in these signaling pathways for good reasons. First, adding some internal dynamics to each agent increases considerably the level of complexity and the number of parameters to tune. Second, it can be computationally costly depending on the formalism used for modeling the signaling pathways inside each agent, such as ordinary differential equations (ODEs) (Clairambault, 2006) or logical formulae (Letort et al., 2018; Varela et al., 2019).

Integrating a proper description of intra-cellular pathways to agent-based models is a challenge, but it becomes crucial when studying the response to a drug that targets specific pathways, even though the effects are often observed at the level of the population (e.g., survival). Indeed, signaling pathways are organized in complex networks encompassing numerous cross-talks and feedbacks. Hence, the deregulation of one specific pathway often leads to non-intuitive effects at the population level.

Mathematical modeling of such complex and intricate networks can help understand and predict experimental results (Cohen et al., 2013; Ferrell, 2015; Kolch et al., 2015; Remy et al., 2015; Abou-Jaoudé et al., 2016). However, the choice of the most appropriate mathematical formalism to model such intra-cellular processes depends on the biological question and the available data (Le Novère, 2015).

Here, we present UPMaBoSS (Update Population MaBoSS), a modeling framework focusing on the dynamics of populations of interacting cells and based on stochastic simulations of a discrete model.

As MaBoSS (Stoll et al., 2012; Stoll et al., 2017)), UPMaBoSS relies on a logical formalism applied to signaling pathways and regulatory circuits. Logical models can be viewed as a coarse grain approximation of more refined and precise modeling approaches, and it proved to be efficient in several studies for which details of the chemical reactions is poorly known (Collombet et al., 2017; Eduati et al., 2017). In this framework, each cell type has its own dynamics and relies on a qualitative model of intra-cellular signaling networks using MaBoSS grammar, thereby enabling probabilistic simulations of cellular models. UPMaBoSS alternates the simulation of intra-cellular models with regular updates of estimates of cell population sizes and environmental signals. These updates are based on the values of key network nodes (receptors and ligands) and processes accounting for cell division and cell death. The whole population can be considered as an heterogeneous cell population, with different cell types, or with cells in different states.

The extension of pathway models constructed in MaBoSS is easy, and the additional computational cost of simulations remains comparable to that of simulations of original MaBoSS models. UPMaBoSS enables the description of complex cellular networks encompassing relatively large numbers of components (up to a few hundreds), interacting through positive and negative influences. Some previous works have already developed a similar algorithm, but based on chemical kinetics (Charlebois et al., 2011; Charlebois and Kærn, 2013).

UPMaBoSS can be used to address biological questions involving interactions between cell types, when signaling pathways are known. As for many approaches considering intra-cellular details, the main application of UPMaBoSS is to explore the effects of perturbations that occur at the level of individual cells in order to understand how these perturbations impact cell populations. Such explorations constitute a first step towards the use of multi-scale modeling for clinical applications (Wolkenhauer et al., 2014; Viceconti and Hunter, 2016).

This modeling approach is illustrated with a simple toy model of cell-cell interaction, and with a case study dealing with the effect of the Tumor Necrosis Factor (TNF) on the cell fate decisions triggered by the engagement of death receptors.

To foster the reproducibility of our results, we provide several notebooks in a dedicated GitHub at https://github.com/sysbio-curie/UPMaBoSS-docker, with examples of models in MaBoSS language, as well as in the more standard SBML-Qual format (Chaouiya et al., 2013). Noteworthy, published logical models of cellular networks (Helikar et al., 2012; Naldi et al., 2018) can be easily adapted and extended by adding cell death, division, and cell-cell interactions. The definition of an UPMaBoSS model then enables the integration of these effects at the population level.

## 2 Materials and Methods

MaBoSS is a tool to simulate continuous time Markov processes on Boolean networks (Stoll et al., 2012; Stoll et al., 2017). It was built as a middle term between the detailed yet complex description of signaling pathways using a chemical kinetics approach and the simpler coarse-grain description using a discrete formalism. To compare these three views (ODE, logical and MaBoSS), models for two network motifs are provided in the Supplementary Material (section 4).

Models of single cell types can each be built using MaBoSS. However, creating a comprehensive model of a dynamic population of these cell types requires following a protocol that can be summarized in two steps: 1) the construction of a single network that encompasses all pathways in all cell types, and 2) the implementation of this network within UPMaBoSS framework. For the latter part, details on the algorithm of UPMaBoSS are given in the Supplementary Section S1.2). For the practical aspects of the UPMaBoSS model construction, we describe the procedure below.

### 2.1 Connecting Cell Population Pathways Into a Single Influence Network

**FIGURE 1:**
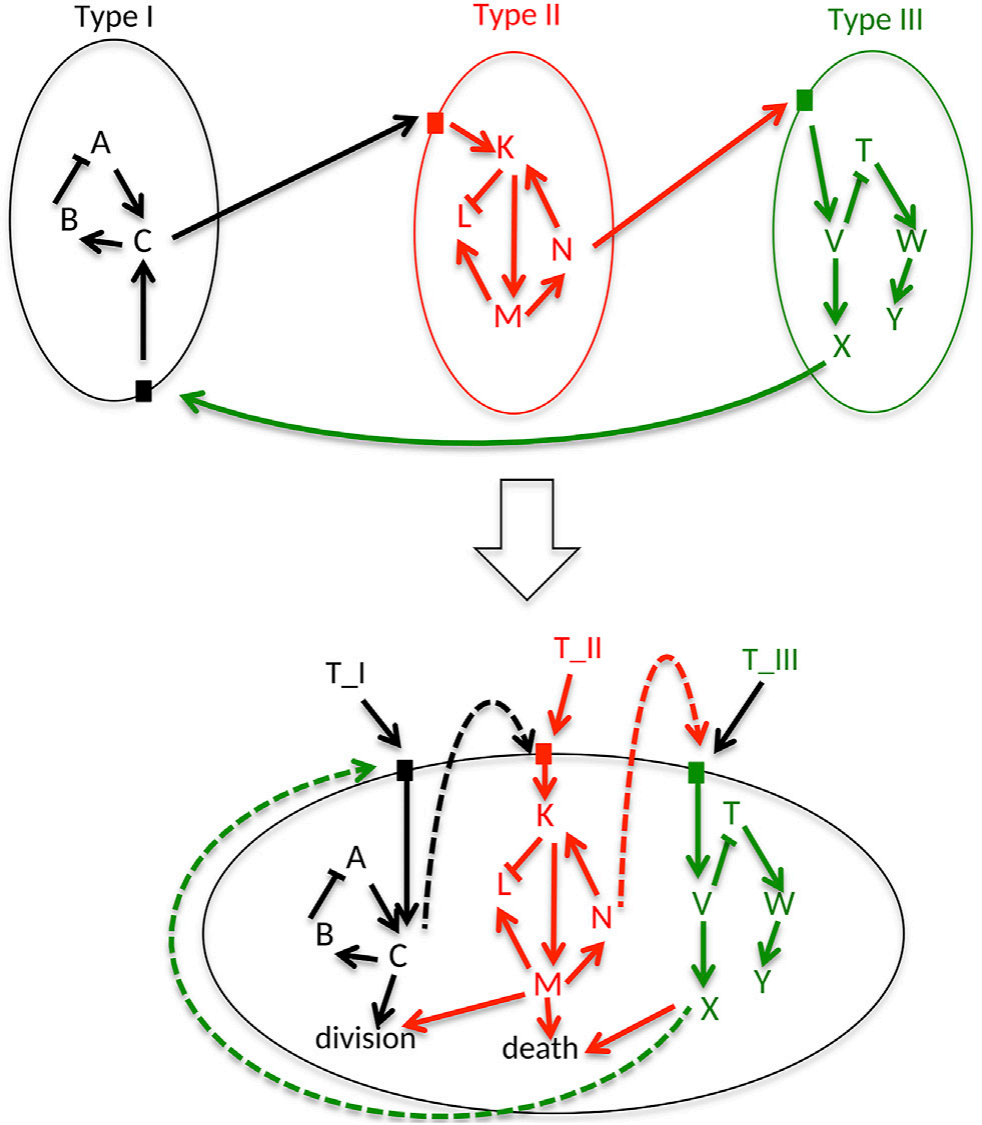
Generic cell. A generic cellular network is constructed by assembling all the signaling pathways that can be activated in different cell types (here 3 cell types are considered: Type I, II, and III), including ligand-receptor interactions. Cells can die, divide or interact through ligand-receptor interactions.

The starting point for an UPMaBoSS model is a set of influence networks that each represents signaling pathways of the cell type they describe. These pathways should contain receptors that are activated by ligands to model properly the interactions between the cell types. The translation of these networks into an UPMaBoSS model requires the following steps, recapitulated in Figure 1:

1 Collect all networks of each cell type and integrate them into a single network (lower panel in Figure 1). Note that it is possible that the obtained integrated network is disconnected. In the case that two pathways belonging to 2 cell types have identical entities, e.g., TGFb, there are two possibilities: either the two networks are merged through the entity or the entity is renamed to specify in which cell type it belongs (i.e., TGFb_Tumor and TGFb_TCell, see Supplementary Section S1.6 for a detailed explanation).
2 Add a node for each cell type (T-Cell, Tumor, etc.), and connect them to the appropriate entities in their associated pathways. For instance, for a protein A activated by B and C in tumor, the network will have ProtB → ProtA, ProtC → ProtA and also Tumor → ProtA.
3 Add two nodes, one that represents cell division and another one that accounts for cell death. The readouts of the model that are related to these phenotypes can then be connected to these new nodes; for example, CyclinB can be connected to division and caspase three to death.

As a result, all possible signaling pathways described in all cell types are gathered into a unique UPMaBoSS model, where the extracellular interactions are represented a feedback loops (Figure 1). This unique network could be understood either as an undifferentiated cell ready to be differentiated into different cell types, or as a population composed of different cell types. This formalism is very flexible and allows the characterization of various cell types present in the micro-environment, as illustrated in the examples in section 3 of the Supplementary Material.

### 2.2 Constructing a Model in UPMaBoSS From an Influence Network

UPMaBoSS relies on MaBoSS software and extends it by adding some important functionalities to model interacting cell populations (Stoll et al., 2012; Stoll et al., 2017).

Every population state is represented by a set of Boolean values associated with the network nodes (including the *division* node, the *death* node, the ligand and the receptors, cell type nodes, etc.). The UPMaBoSS framework enables the computation of the time-dependent probabilities of states, allowing an interpretation of the dynamics at the cell population level, considering that:

(Number of cells in state S)=(Number of cells)×(Probability of S)

The definition of the model components assumes that each node of the network represents a gene, protein, complex, phenotype or cell type. The UPMaBoSS model gathers the pathways and entities potentially active in the different cell types. It is derived from an influence network by applying the following procedure:

• For every node, except receptors, two transition rates are defined, rate_up for activation and rate_down for inhibition, with triggering rules formulated in the language of MaBoSS (see the Results section for an example of the grammar used for writing logical rules and transition rates).
• For receptors, the rates associated with the update of their state must contain term(s) that depend on the population state probabilities.
• The initial conditions for all the entities of the model need to be set with the same formalism as in MaBoSS: a probability can be associated with each node of the model (e.g., [A]. istate = 0.2 [0], 0.8 [1] means that the node A will start with 80% of the trajectories with A in state 1) or to a vector of nodes (e.g., [A,B]. istate = 0.2 [0,0], 0.5 [0,1], 0.1 [1,0], 0.2 [1,1] means that 20% of the trajectories will start with both nodes at 0, 50% with B active only, 10% with A active only and 20% with both nodes active).

**FIGURE 2:**
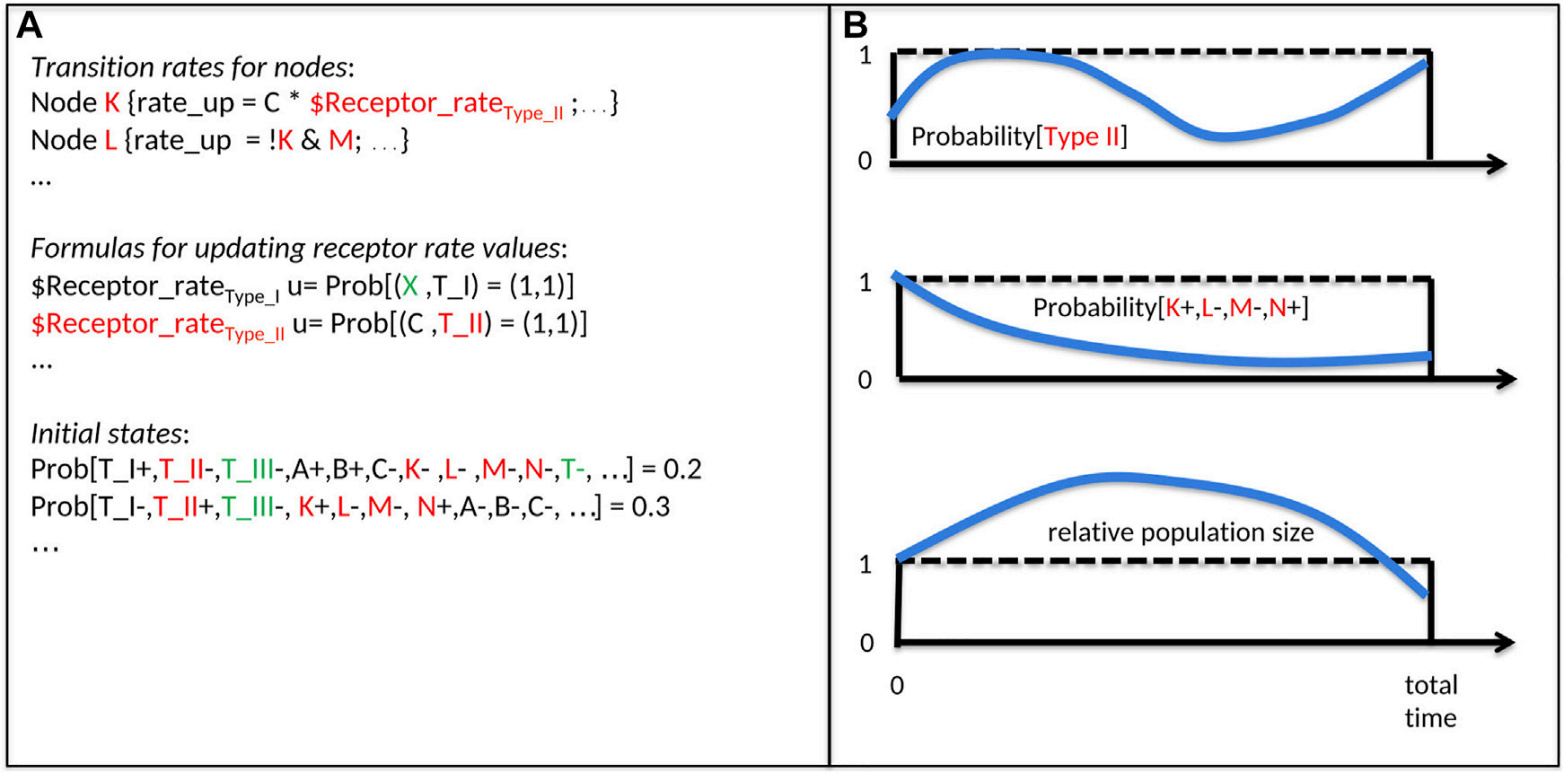
Inputs and Outputs of an UPMaBoSS model. The notation is related to Figure 1: A, B, C, K, L, M represent genes/proteins; T_I, T_II, T_III represent cell types. **(A)** Inputs of UPMaBoSS: Transition rates for nodes: for each node (here K and L of Figure 1), a logical rule, the rate up and rate down are written; Formulas for updating receptors rates values: the update rules, starting by u = …, depend on the population state and regulate the value of the external variable $Receptor_rate of cell type I and II; and initial states: they can be defined such that cell types, proteins, etc. can be characterized as present (+) or absent (−) with a probability for this model state to be active initially. Colors correspond to cell types of Figure 1. Note that names starting with a $ correspond to external variables, specific to MaBoSS/UPMaBoSS, listed in bnd file, set up in cfg file and updated in upp file. **(B)** Ouputs of UPMaBoSS: time-dependent probabilities of cell types (upper panel, example of cell type II from Figure 1), with the corresponding model states (middle panel), and the time-dependent population size (lower panel).

For each time step, UPMaBoSS computes the relative population size (with respect to the initial size), and the distribution of state probabilities in the population (including the status of *death*, *division*, and cell type nodes) (Figure 2).

UPMaBoSS launches several consecutive MaBoSS runs (the number of runs being defined by the user). At the end of each run, the population is updated synchronously: new model states are produced according to the parameters influencing the population status (death, division, receptor activity), setting a novel initial condition for the next MaBoSS run (see section 1 of Supplementary Material for more details).

### 2.3 Tuning Parameters

An UPMaBoSS model contains a set of parameters that need to be calibrated. They can be separated into two families: parameters with a biological interpretation versus those modulating the simulations.

The first family of parameters include the rates of activation or inactivation of a variable, which can be derived from experiments. They can correspond to the mean time necessary to achieve transcription, (de)phosphorylation, synthesis or degradation. These transition rates can be separated into fast or slow variables. If such information is not available, the default value 1.0 is used. Other parameters can account for the initial conditions or the duration of the experiment (total time of simulation is reached when (total time) = (number of steps) × (length of MaBoSS run)).

The second family of parameters corresponds to the number of trajectories to include in the computation, and the length of one MaBoSS run (ensuring that transitory behaviors are observable, see an example below).

An exhaustive list of these parameters is provided in the Supplementary Section S1.5, including default values and guidelines for choosing their values. In some cases, a sensitivity analysis may be needed to select the appropriate range of parameter values. We provide an example with a variation of the length of one MaBoSS run (*max_time*) and analyze its impact on the expected results in a python notebook included in the GitHub folder (https://github.com/sysbio-curie/UPMaBoSS-docker/tree/master/CellFate).

## 3 Results

We illustrate the use of UPMaBoSS with two examples, the first one focusing on cell-cell interactions, and the second one extending a published model of cell fate decision in response to the activation of death receptors (Calzone et al., 2010).

### 3.1 Toy Model

First, we present a simplified model to highlight important dynamical differences occurring when considering a unique cell model to represent a population of non interacting cells (MaBoSS simulation) versus considering a dynamic population of interacting cells (UPMaBoSS simulation). This simple model implements a differentiation mechanism leading to two cell types, *T*1 and *T*2. This differentiation process is initiated by a trigger *I*. When *I* activates *A*, *A* is able to drive the *T*1 cell type differentiation. At the same time, *A* is also able to activate the ligand *L*, which itself activates a receptor *R* leading to *T*2 cell type differentiation, but only in the absence of *A*. The activation of *T*2 is then irreversible (no degradation rate).

**FIGURE 3:**
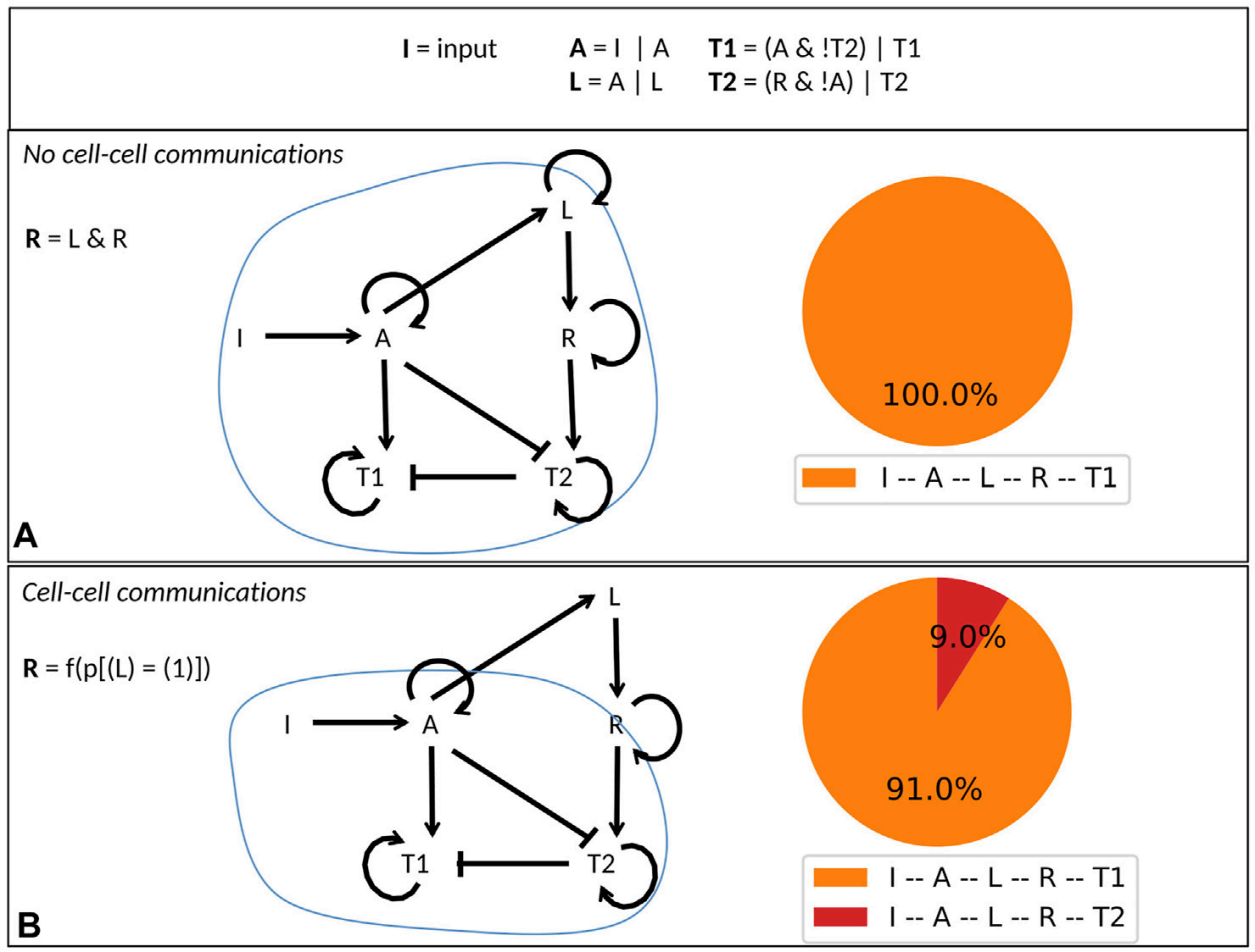
Simple cell differentiation model. **(A)** Definition of the toy model with logical rules (upper panel) and conditional rule for R depending on the value of the external parameter innerOn. If innerOn is equal to 1, then A is able to activate L in all cells (middle panel). If innerOn is set to 0, then the value of R will depend on the population status of L (lower panel). **(B)** Model simulations of the two cases: when innerOn = 1, only T1 cell type can be reached; when innerOn = 0, a proportion of cells can differentiate into T2 cell type.

We compare the behavior of a population of independent cells with that of a population of interacting cells (through ligands and receptors). The difference between these two situations lies in the logical rule associated with the receptor variable *R* (*R* = *innerOn* ? *L* : *outerL* in the model file, which reads as if *innerOn* = 1 then *L* else *outerL*). In the case of a single cell model, when cell-cell interaction is not considered, *R* is activated by *L* (*innerOn* = 1 in the parameter file) (Figure 3A). In the case of a population model, when cells can interact, *R* is activated by a function of the probability for *L* to be active (*innerOn* = 0 and *outerL* = 5*p [(*L*) = (1)] defined in the population file) (Figure 3B).

When a population of independent cells is considered, *A* is always present when *R* is active, which continuously inhibit *T*2, ultimately leading to the *T*1 phenotype (Figure 3A).

When a population of interacting cells is considered, *R* is updated according to the population state of *L*. Consequently, *R* can be activated in some cells independently of the activity of *A*, allowing differentiation in the *T*2 cell type with a non-zero probability (Figure 3B). In other words, with *I* active, we obtain about 87% of T1 cells (where *I*, *A*, *L*, *R*, and *T*1 are active) and 13% of T2 cells (where *I*, *A*, *L*, *R*, and *T*2 are active). *A* can eventually reactivate in those cells, but we assumed the T2 phenotype to be irreversible (self loop).

This simple example highlights a mechanism occurring only when cells are allowed to interact. When integrating data into a model, fitting parameters, or constructing a Boolean model from experimental data, these considerations might be of importance.

We provide a Jupyter notebook for this example, including the UPMaBoSS model (with the three corresponding files) and an explanation of how to build an interacting cell population model from a standard cell model [defined with the bnet format (Müssel et al., 2010)] (see GitHub at https://github.com/sysbio-curie/UPMaBoSS-docker/tree/master/ToyModelUP).

### 3.2 A Model of Cell Fate Decision

This case study provides an example of the use of UPMaBoSS to model the response of a cell population to different drug treatments. In this respect, the model integrates pathways controlling cell proliferation and death, which has a direct impact on the size of the cell population.

#### 3.2.1 Description of the Model

We start with a model initially built to understand how the same signal can lead to three different cell phenotypes depending on the cellular context. The activation of the death receptors TNFR or Fas by their respective ligands can trigger a cascade of events leading to either survival with the activation of NF*κ*B pathway, or to non-apoptotic cell death (referring to necrosis or to a programmed necrosis called necroptosis) with the loss of ATP, or to apoptosis with the cleavage of caspase 3 (Calzone et al., 2010). This generic model was built on the basis of data collected from the literature, focusing on the main components influencing the cell fate decision between death and survival. The model can be found in the GINsim repository: http://ginsim.org/node/227. The original analysis explored which components contribute to each phenotype, as well as the cross-talks between the three pathways, enforcing mutual exclusion of the three alternative fates.

**FIGURE 4:**
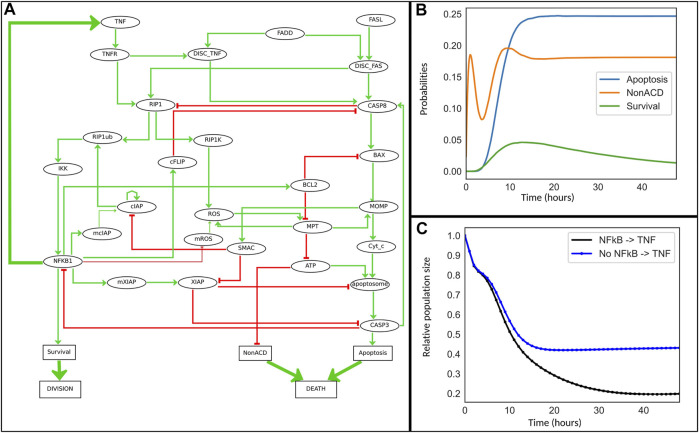
Cell fate model for TNF*α* resistance. **(A)** This model is an extension of the model reported in (Calzone et al., 2010). Some nodes representing the mRNA of cIAP, ROS and XIAP family members have been added. The ellipsoid nodes represent genes, mRNA, proteins, or complexes, while the rectangular nodes denote phenotypes. Green and red arrows represent positive and negative influences, respectively. The thick green arrows denote activating interactions added to the initial model: a feedback from NF*κ*B to TNF*α* encodes the ligand-receptor activation, while the “Division” and “Death” nodes have been introduced specifically for UPMaBoSS population updates. **(B)** Simulation of the cell fate model with MaBoSS for 48 h. **(C)** Simulation of the model with UPMaBoSS: temporal evolution of population sizes with (black) and without (blue) the TNF paracrine signaling.

In the present study, we extend this analysis by considering the impact of the timing and duration of TNF treatments. To this end, a feedback from NF*κ*B pathway to TNF*α* was added to the model (Figure 4A). Indeed, it has been showed that TNF*α* is a target of NF*κ*B, and that constitutive activation of NF*κ*B leads to systemic inflammation through TNF*α* activation (Shakhov et al., 1990; Drouet et al., 1991; Liu et al., 2000; Dong et al., 2010). We further decided to focus on the role of TNF*α* and thus kept Fas OFF for all our simulations.

We explored the effect of sequential treatments of TNF*α* at a cell population level. Interestingly, several studies showed that prolonged treatments of low doses of TNF can lead to resistance in prostate cancer patients (Smyth et al., 2004), and that TNF exhibits a dual impact on tumor progression: at low doses, it triggers angiogenesis (Wang et al., 2017), whereas at high doses, it induces cell death, mainly through necrotic effects (Bertazza and Mocellin, 2010).

#### 3.2.2 Biological Questions

In a first *in silico* experiment, we simulated the model for a period corresponding to 48 h of cell culture (unit time set to 1 hour). Experimentally, there is no consensus for time duration of TNF effect *in vitro*; nevertheless, key events are known to require over 24 h to occur (Udommethaporn et al., 2016), which justifies the choice of 48 h. Indeed, in our simulations (Figure 4), stability is reached after 48 h. For a transient treatment of TNF, we considered a TNF half-life of 4 h (degradation rate of 1/4). Although TNF degradation rate varies extensively depending on experimental conditions, 4 h seems a reasonably small interval compared to 48 h.

We first simulated the model with MaBoSS, which is particularly important in order to define the time step for each MaBoSS run when using UPMaBoSS. Indeed, the chosen time window (i.e., max_time) must be such that the population is in a transient state. The best value for the parameter max_time turned out to be around 1 h (just before the peak of activation of Figure 4B). To simulate the population dynamics, we proceeded to compute 48 MaBoSS runs with max_time equal to one for each run. We further studied the sensitivity to this parameter and included online (Jupyter notebook *TimeStepDependency.ipynb*, https://github.com/sysbio-curie/UPMaBoSS-docker/tree/master/CellFate).

In the MaBoSS simulation, we noticed that non-apoptotic cell death first decreases, before increasing to reach a steady state solution after *t* = 15 h. This dynamics is due to the activity of ATP, itself dependent on that of RIP1. RIP1 increases until CASP8 is activated and able to inhibit it. It takes longer to activate CASP8 than RIP1. This behavior is typical of incoherent feedforward loops (Jin, 2013).

For this biological application, we focused on two questions:

1 What is the effect of the feedback from NF*κ*B to TNF*α* at the population level when treated by a transient activation of TNF*α*?
2 What is the effect of TNF*α* sequential treatments on the population dynamics?

**FIGURE 5:**
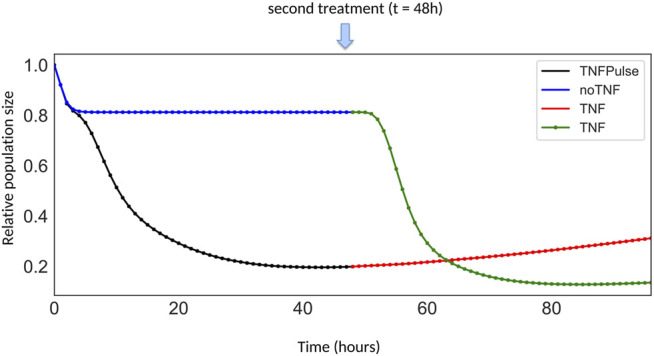
Growth curves for different TNF treatment scenarios. The first scenario corresponds to the simulation of cells initially treated by a pulse of TNF (black segment), followed by a constitutive TNF treatment at *t* = 48 h (red segment). The other scenario corresponds to the simulation of cells initially untreated (blue segment), but receiving a constitutive TNF treatment at *t* = 48 h (green segment).

To address the first question, two model variants were considered: with and without the NF*κ*B → TNF paracrine loop, with a transient TNF treatment (Figure 4C). For the second question, we selected the model with the paracrine loop and further studied the two following scenarios in which cells are treated with: 1) a transient TNF treatment at time 0 (“TNF Pulse”), followed by a constant TNF treatment at 48 h (“TNF”) and 2) no TNF treatment at time 0 (“NoTNF”), followed by a constant TNF treatment at 48 h (“TNF”) (Figure 5).

#### 3.2.3 TNF Treatments in Wild Type Conditions

Simulations of the temporal evolution of cell populations are displayed in Figure 4C in presence or absence of the feedback and in Figure 5 for two different TNF treatment scenarios.

In Figure 4C, following a pulse of TNF at *t* = 0, the comparison of population growth curves in the absence (blue curve) or in the presence (black curve) of the feedback from NF*κ*B to TNF*α* indicates that the TNF paracrine loop leads to a decrease of the population size (from 43% to 20% of the initial population size of 100%) confirming the stronger effect of the feedback in TNF-treated cells.

Remarkably, when a sustained treatment is applied, the impact on the population size differs depending on whether the population has already been treated or not, in a non-intuitive way. Indeed, as shown in Figure 5, after 48 h, the population initially untreated (blue + green curves) decreases faster than the cell population initially treated with a pulse of TNF (black + red curves). This difference could be interpreted as a resistance mechanism: cells that have already been exposed to TNF, even transiently, do not respond as well to a second TNF treatment compared to cells that have never been treated with TNF. Noteworthy, this “resistance” results purely from network dynamics, in the absence of any genetic modifications and could be related to network motifs, as proposed in (Charlebois et al., 2014; Camellato et al., 2019). Intuitively, this behavior arises from the mutual inhibitory connections between survival and death pathways. Indeed, the first TNF treatment selects the cells that have activated genes of the survival pathway, and therefore cannot activate their death pathway upon a second TNF treatment. In the following section, we further investigate which parts of the network contribute to drug resistance by comparing wild-type and various mutant simulations.

#### 3.2.4 TNF Treatments in Mutant Conditions

In the clinics, the various mutations found in patients may affect the efficacy of the response to treatments. To explore the potential roles of the different model components in the observed TNF resistance, we simulated the effects of all possible single mutants (Jupyter notebook *CellFateModel_upmaboss* at https://github.com/sysbio-curie/UPMaBoSS-docker/tree/master/CellFate).

For each single mutant, we measured the population ratio at t = 96 h for four possible scenarios: “No TNF” + “No TNF” (control, dashed green curve), “No TNF” + “TNF” (late treatment, plain green curve), “Pulse of TNF” + “No TNF” (single early treatment, dashed red curve), and “Pulse of TNF” + “TNF” (consecutive treatments, plain red curve).

**FIGURE 6:**
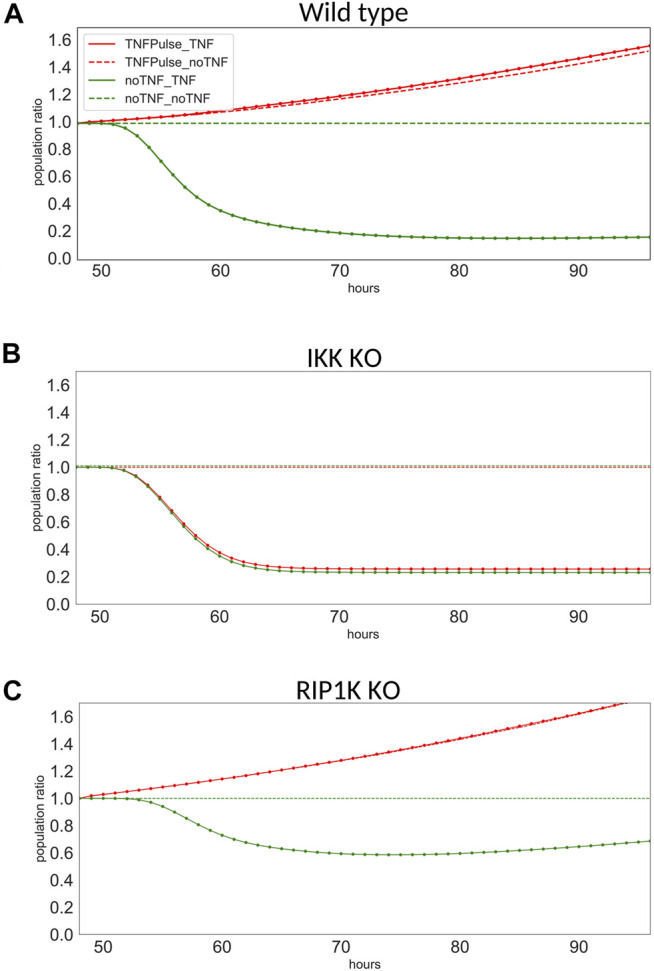
Population ratio at from *t* = 48–96 h for three models. Population ratios for the four conditions in **(A)** wild type, **(B)** IKK knock-down, and **(C)** RIP1K knock-down.

Here, we focus on the results obtained for three genetic backgrounds: wild type, IKK knock-down, and RIP1K knock-down (Figure 6). For sake of simplicity, we focus on the response after *t* = 48 h, and to ease the comparison, we normalized the population ratio found in Figure 5. Note that two conditions were added to those shown on Figure 5, where only two of the four scenarios were simulated (i.e., we added the cases when there is no treatment after 48 h, no matter what the cells receive at *t* = 0, dashed lines).

The wild type model clearly exhibits a resistance: when the cells have received a first treatment, they do not respond, whether they receive a second treatment or not (Figure 6A, for TNFPulse_TNF and TNFPulse_noTNF). The resistance effect is lost for IKK knock-down (Figure 6B), as the mutated cells respond to the treatment whether they received a first treatment or not (plain and dashed lines coincide). In the case of the RIP1K knock-down, the decrease of the population size after TNF treatments is milder than for wild type model, but the resistance mechanism is still observed (Figure 6C).

IKK belongs to the NF*κ*B pathway that induces survival. This pathway introduces a positive feedback at the cell population level, which may explain why IKK knock-down shuts down the resistance to TNF by blocking this feedback loop. RIP1K induces non-apoptotic cell death by blocking ATP, which explains the reduction of the apoptotic effect of TNF observed for the RIP1K knock-down.

The effects of double mutations can also be simulated. However, for our example, the double mutant simulations do not result into additional insight because the single mutations are already informative.

All results and figures of this analysis can be reproduced with the Jupyter notebook provided online in the folder *usecases* in the CoLoMoTo Docker image (https://colomoto.github.io/colomoto-docker/), as well as on github: https://github.com/sysbio-curie/UPMaBoSS-docker/tree/master/CellFate).

## 4 Discussion

UPMaBoSS is a novel modeling framework enabling the exploration of cell population dynamics. It considers the division, the death and interactions of cell populations and relies on stochastic simulations with regular synchronous updating of cell populations.

Using a simple toy model, we first showed that the results of the simulations are different if we consider a homogeneous, non-interacting cell population compared to a dynamic population of interacting cells. We further applied our approach to a model of TNF-induced cell fate. We show that the paracrine loop involving NF*κ*B enhances cell death. Surprisingly, our simulation revealed an intriguing resistance mechanism: once the cells have been treated by TNF transiently, they can resist to a second treatment, an effect not attributable to genetic selection. One limitation of our approach lies in the setting of population update time. However, the case study presented here suggests that results are moderately sensitive to changes of this parameter within a reasonable interval. Finally, an application of this modeling framework to a concrete example describing events of the immunogenic cell death was recently published (Checcoli et al., 2020). In this study, the main steps of the immunogenic cycle and the relative timing of the events were reproduced: the parameters were chosen so that some processes such as the migration of the dendritic cells to the tumor micro-environment would take the expected time (around 12 h).

Simulations and predictions obtained with UPMaBoSS could be validated experimentally using different techniques: the probabilities of the nodes corresponding to proteins/mRNA can be compared to their experimental relative concentrations (measured by western blot, immunofluorescence, qPCR, etc.). This can be done by considering positive and negative controls, since what we are most interested in with this approach is to compute the change of probabilities between two experiments or 2 cell conditions rather than compare exactly the model probabilities with quantitative experimental measures. This comparison can be facilitated by normalizing experimental measures between 0 and 1. The probabilities of the network states could be validated using flow cytometry or microscopic images, using thresholds that separate active/inactive states. Single-cell transcriptomic (with CRISPR/Cas9 genetic engineering) and flow cytometry (on cell population markers), in principle, can be used to estimate the sizes of different cell sub-populations: once thresholds have been applied on the quantitative markers defining cell populations, the experimental data can be translated in terms of cell sub-populations.

There are, of course, a number of limitations with our framework. First, UPMaBoSS is not meant to answer precise biological questions on diffusion, localization, or drug dosage. More quantitative modeling frameworks would be more appropriate to address such questions. However, previous studies suggested that the presence, rather the precise localization, of immune infiltrates in tumors constitute good prognostic biomarkers (Baxevanis et al., 2019), which could be modeled with UPMaBoSS.

Another limitation is that UPMaBoSS is not optimal to model metabolic networks, which involve reactions consuming reactants. In practice, metabolites could be included in UPMaBoSS models, in particular if they play a role in signaling pathways. Metabolites are then represented by Boolean nodes, implying and requiring a proper discretization of their levels (multilevel variables can then be encoded by multiple Boolean nodes).

We think that this approach is appropriate to model many processes in cell biology, including cell differentiation, innate/adaptive immune system activation, cancer micro-environment, and tissue homeostasis. The fact that the spatial dimension is not taken into account might appear as a limiting factor for clinical applications. In this respect, it is possible to extend the model using PhysiBoSS, a multi-agent modeling tool in which each agent is a cell running an intracellular MaBoSS model, but which requires to tune more parameters (Letort et al., 2018).

In the future, we plan to apply UPMaBoSS to model the effect of the micro-environment on cancer cell fate. UPMaBoSS simulations are based on consecutive runs of MaBoSS. While MaBoSS generates tens of thousands trajectories, UPMaBoSS parses and writes probability distributions for each population updates. Consequently, computational cost increases moderately (less than an order of magnitude) compared to prior MaBoSS models. Moreover, because UPMaBoSS can be run directly on a conda environment or within a simple pair of python and C++ scripts, simulations can be launched on High Performance Computers, with a natural parallelization for tasks such as parameter variations and gene mutations.

Finally, on the practical side, the implementation of UPMaBoSS in a docker image and in a conda package should greatly facilitate its use, whereas the reproducibility of the analyses can be enforced by the use of Jupyter notebooks.

## Data Availability

The original contributions presented in the study are included in the article/Supplementary Material, and on a GitHub repository (https://github.com/sysbiocurie/UPMaBoSS-docker). Further inquiries can be directed to the corresponding authors.
