# Supplementary material for: UPMaBoSS: A Novel Framework for Dynamic Cell Population Modeling
Source: Front Mol Biosci. 2022 Mar 2;9:800152. doi: 10.3389/fmolb.2022.800152 (PMC8924294; doi:10.3389/fmolb.2022.800152)
Supplement: Supplementary file 1 [file Presentation1.pdf]

Supplementary Material for ”UPMaBoSS: a novel framework for dynamic cell population modeling”

1 DESCRIPTION OF UPMABOSS

UPMaBoSS is provided with both perl and python scripts that produce multiple runs of MaBoSS (or "steps"), in order to provide a modelling framework at population level. UPMaBoSS needs three plain text files with specific extensions: a *bnd* file for the model description (**b**oolean **n**etwork **d**escription, which lists all the logical rules and associated external variables for each node), a *cfg* file for the **c**on**fig**uration of the parameters (which includes both model and simulation parameters such as time of simulations, number of trajectories) for each MaBoSS run, and an *upp* file that defines the different parameters describing the cell population. Note that all nodes must be set as external (in the *cfg* file).

1.1 The *upp* file

This file needs to contain:

- A line defining the model node (present in the *bnd* file) that launches cell division, *e.g.*
  `division = Division_Model_Node;`
- A line defining the model node (present in the *bnd* file) that launches cell death, *e.g.*
  `death = Death_Model_Node;`
- A line defining MaBoSS executable name, *e.g.*
  `MaBoSS = MaBoSS_custom_name;`
- A line defining the number of steps, *e.g.*
  `steps = 16;`

This file can also contain line(s) defining external variable(s), used in the *bnd* file, which are updated at each step. Each of these lines should start with the name of an external variable, followed by the update operator `u=` and an expression using the same operators than in MaBoSS language:
`+,-,*,/,AND,OR,XOR,NOT,(?:)`.
Probabilities of different states can be inserted, using the following syntax:
`p[(node1,node2,...)=(1,0,...)]`.
Two keywords can be inserted: `#rand` representing a random number between 0 and 1 and `#pop_ratio` representing the population ratio. For instance:

```
$ExtVar1 u= 5*(p[(Node_A,Node_B,Node_C) = (0,1,0)])
```

Note that each of these external variables needs to be also defined in the *cfg* file, in order to run UPMaBoSS.

1.2 Algorithm

The algorithm is illustrated in Figure S1.

For the step 0, UPMaBoSS runs MaBoSS according to the *bnd* and *cfg* files.

For the following steps, UPMaBoSS runs MaBoSS according the *bnd* file and a new *cfg* file (named `cfg_file_step_n.cfg`, where `cfg_file` is the name of the initial *cfg* file). For that, an updated

probability distribution is computed from the final probability distribution of the step $n-1$ in the following way:

1. Set to 0 each probability whose state has the death node active.
2. Double each probability whose state has the division node active.
3. Define the population ratio of step $n-1$ as the sum of all probabilities (after applying 1 and 2).
4. Set division node to inactive for every state in the probability distribution.
5. Divide all probabilities by the population ratio.

The initial *cfg* file is copied into a new file `cfg_file_step_n.cfg`. The updated probability distribution (above) is injected in this new file as the new initial condition. The values of every external variable in the upp files are computed according to this updated probability distribution. These new values of external variables are injected in the new *cfg* file `cfg_file_step_n.cfg`.

1.3 Description of output files

For the executable version of UPMaBoSS (in perl), a cfg file is generated for each MaBoSS run. In addition, two files are provided: `Model_PopR.csv` and `Model_PopProbTraj.csv`.
`Model_PopR.csv` contains the population ratio at each time step.
`Model_PopProbTraj.csv` contains network state probabilities at each time step.

1.4 Choice of time step length

The `max_time` value in the *cfg* file is considered as the "time step", because UPMaBoSS updates the population model after a MaBoSS run of this length. Therefore, this value needs to be carefully chosen:

- The time step should be smaller than the first transient effect.
- The time step should be large enough so that this transient effect had already been initiated.

Therefore, it is strongly suggested to first produce a single run of MaBoSS, using the *bnd* file and a modified *cfg* file that has a much longer `max_time`, in order to estimate these lower and higher limits of the "time step" described above. Then, time step sensitivity within its lower and higher limits should be studied (see the example below).

1.5 Parameters to be set for a simulation

Parameters that have a biological interpretation

- Activation/inhibition rates: numerical values put in the formulas for `rate_up` and `rate_down` in the *bnd* file. They represent the speed of activation/inhibition of the associated node. A possible interpretation of this value is 1 divided by the time needed for the transition: $1/$time. The suggested default value is 1.0. They are related to the unit of time used for the length of time steps defined below, e.g., if the unit of time steps is [hours], the rates are expressed in unit $[1/\text{hours}]$.
- Time length of simulation. This value is set by the number of steps given in the *upp* file. The length of the simulation corresponds to this number of steps multiplied by the length of time steps (see below) defined as the length of MaBoSS simulation (`max_time` in the *cfg* file). As default, when activation/inhibition rates are close to 1, we suggest to take 10 steps for a time step length of 10.
- Initial conditions. The initial condition is set as a probability distribution over the set of network states. As default, we suggest to take a random probability distribution, where all network states have an

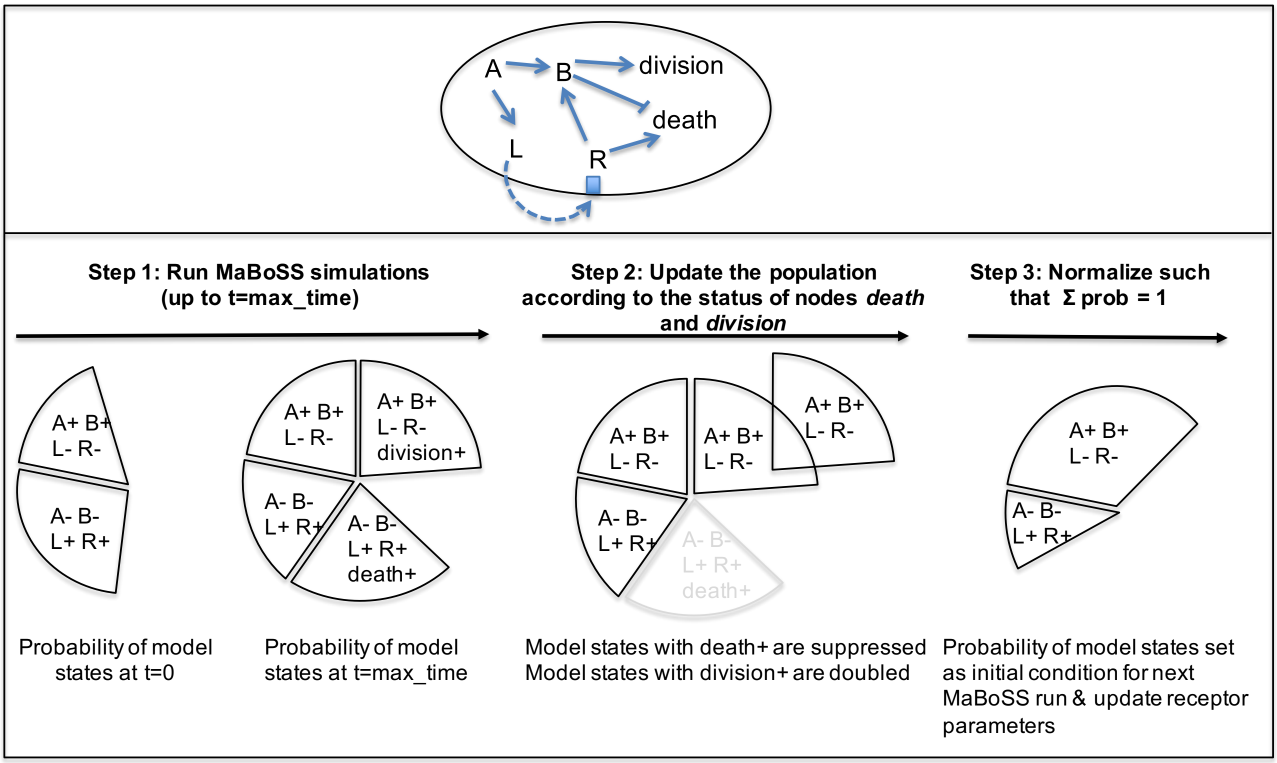

Figure S1. Key steps of UPMaBoSS simulations. (Top panel) Simple, illustrative model, where A can activate B and release L (ligand) outside the cell, which activates R (receptor) on the membrane of the same cell; R can, on one hand, activate signaling pathways leading to cell death, and on the other hand, trigger pathways leading to cell division and inhibition of death through the entity B. (Bottom panel) For the model shown in the top panel, UPMaBoSS algorithm iteratively applies the three following consecutive steps. *Step 1*: MaBoSS is run for a given initial condition and time length (only some selected model states are shown here: the state [A+,B+,L-,R-] means that only A and B are at 1 and L and R are at 0, the size of the pie portion corresponds to its probability); for some states, this leads to the activation of the nodes division or death. *Step 2*: After the MaBoSS run, state probabilities are updated by removing those that have the death node active and doubling those that have the division node active; the size of the population is multiplied by the sum of these new probabilities, which are then normalized. *Step 3*: A factor in the transition rates of the receptors is updated with the newly computed probabilities; the next MaBoSS run starts with the normalized probabilities as initial condition. The number of runs n is defined by the user and this sequence of three steps is thus repeated n times.

equal probability. When modeling a specific pathway that represents *in vitro* experimental data, all node could be set at zero except the one that activates the pathway.

- Update formula for the external variables related to the activity of receptors. This is given by the equation, in the *upp* file, that describes the update of an external variable in term of state probabilities. Any formula using $+, -, *, /, \log, \exp$ can be used. For an external variable (eg `$outerL`) describing an activation rate of a receptor by a ligand (L), we suggest to use `$outerL u= p[(L) = 1]`.
- Time tick. This value is set in the file (`time_tick`). It represents the length of time windows on which probabilities are estimated. Although it is preferable to use small values, a too small value will produce non-continuous probability trajectories. This value should not be larger that the time window on which time dependent experimental values are measured (every hour, day, etc.). The suggested default value is 0.1.

Modeling parameters

- Number of MaBoSS trajectories. This parameter, set as `sample_count` in the file, controls the quality of simulation. It is recommended to increase this value if the results depend too much on the seed of the random generator. The suggested default value is 10000
- Seed of random generator. This parameter, set as `seed_pseudorandom` in the *cfg file*, allows to test the stability of the results by changing its value. There is no suggested default value.
- Length of time steps between each update. This is set in the *cfg* file by the `max_time` value, see the discussion above. The unit of time is the inverse of the unit of activation/inhibition rates. The suggested default value is 10.

1.6 MaBoSS grammar for merged pathways with common nodes

It may happen that that the same entity appears in different cell types. Such cases can be implemented in different ways, according to two main situations:

1. when the entity is measured globally over the whole micro-environment, no matter where the entity is located (e.g., transcriptomics, western blots, etc.). In this case, the rates of transitions associated to each cell type should be linked to the respective cell type node and merged into a single rule: *rate_up = (NodeTypeA ? ValueAInTypeA : (NodeTypeB ? ValueIntypeB : 0))*, which can be read as if NodeTypeA (e.g., T cells) is true, then the rate_up will take the value ValueAInTypeA, otherwise, if NodeTypeB (e.g., Tumor) is true, then the rate_up will take the value ValueAInTypeB, and if both are wrong, then the rate_up will take the value 0.
2. when the entity is measured in a specific cell type, as it is done in flow cytometry experiments, then the name of the entity should be associated to the cell type, e.g., TGFb will be renamed to TGFb_TCell and TGFb_Tumor.

2 APPLICATION: POPULATION MODELING OF CELL FATE MODEL

2.1 Model parameters

The population version of TNF induced cell-fate is constructed in the following way:

1. We used a published model, translated in MaBoSS language (available in MaBoSS webpage: https://maboss.curie.fr/).
2. We added a node "Division", induced by "Survival" node, that has an activation rate of 1/12 (half a day).
3. We added a node "Death", induced by "Apoptosis" or "NonACD" nodes, that has an activation rate of 1 (one hour).
4. We fixed the time step to 1 hour, because the transient activation of death has its first maximum at 1 hour.
5. For implementing TNF production by NFκB, we added an external variable $TNF_induc. This variable is updated according to the probability of having NFκB active and Death inactive.
6. We launched UPMaBoSS.pl with 48 steps, upon different conditions (see below).

We consider the following conditions:

- Transient TNF (initial state with TNF active, TNF degradation rate of $1/6$).
- Transient TNF with no cell-cell interaction (no induction of TNF through NFκB).

- No TNF (TNF inactive initially).
- Permanent TNF ($TNF_induc at 20 in the initial *cfg* file), starting from the asymptotic state from the previous UpPMaBoSS run (transient TNF or no TNF).

2.2 Time step sensitivity

A Jupyter notebook (`TimeStepDependency.ipynb`) describes the effect of changing the max_time for two conditions when TNF is ON (TNF) and when TNF is OFF (NoTNF). The different population ratios are compared for the values 1 (the value used for our example), 0.5, 0.33, and 0.25. This example is available within the CoLoMoTo Docker image at https://github.com/sysbio-curie/UPMaBoSS-docker.

3 POSSIBLE EXTENSIONS OF THE MODELING SCHEME WITH UPMABOSS

3.1 Sensitive induction of receptors

Situation: a receptor `R` is extremely sensitive to the activation by its ligand(s) `L`.

Solution: use a non-linear function, e.g.:

In the *bnd* file:

```
node R {
  rate_up = -log(1-$probL) ;
  rate_down=0;
}
```

In the *upp* file:

```
$probL u= p[(L = 1)]
```

3.2 Multi-level description

Situation: a node `A` has more that two possible values.

Solution: replace the node `A` by several nodes (number of levels $-$ 1), adapt the transition rates, e.g. for 4 levels:

In the *bnd* file:

```
node A_1 {
  rate_up = (B AND C) ? 30.0 : 0.0;
  rate_down = (NOT A_2 AND NOT (B AND C)) ? 30.0 : 0.0;
}
node A_2 {
  rate_up = (A_1 AND D AND F) ? 30.0 : 0.0;
  rate_down = (NOT A_3 AND NOT (D AND F)) ? 30.0 : 0.0;
}
node A_3 {
  rate_up = (A_2 AND G AND H) ? 30.0 : 0.0;
  rate_down = (NOT (G AND H)) ? 30.0 : 0.0;
}
```

The initial condition should be carefully defined, in order to avoid impossible states (e.g., active A_3 and inactive A_1), e.g., in the *cfg*:

```
[A_1,A_2,A_3].istate = .3 [0,0,0] , .2 [1,0,0] , .4 [1,1,0] , .1 [1,1,1] ;
```

3.3 Spatial organization

Situation: cells should be considered spatially.

Solution: add nodes that represent compartments.

In the *bnd* file, for three aligned compartments (C0, C1, C2) represented by the two nodes C_1 and C_2: cell in C0, $(C\_1, C\_2) = (0,0)$; cell in C1, $(C\_1, C\_2) = (1,0)$; cell in C2, $(C\_1, C\_2) = (1,1)$:

```
node C_1 {
  rate_up = (B AND A) ? 10.0 : 0.0; // Condition for moving from C0 to C1
  rate_down = (NOT C_2 AND NOT (B AND A)) ? 10.0 : 0.0;
}
node C_2 {
  rate_up = (C_1 AND D) ? 1.0 : 0.0; // Condition for moving from C1 to C2
  rate_down = (NOT D) ? 1.0 : 0.0;
}
```

3.4 Extracellular chemical diffusion

Situation: a ligand needs some time and condition(s) to diffuse in extracellular matrix to activate its receptor.

Solution: add diffusion states to the produced the ligand.

In the *bnd* file, for a ligand L that needs two steps (L -> L_d1 -> L-> L_d2) to reach its receptor (mean migration time of $1/5.0 + 1/5.0$, mean degradation time of $1/0.1$:

```
node L {
  rate_up = A ? 1.0 : 0.0;
  rate_down = A ? 0.0 : 1.0;
}

node L_d1 {
  rate_up = L ? 5.0 : 0.0;
  rate_down = 0.1 ;
}

node L_d2 {
  rate_up = L_d1 ? 5.0 : 0.0;
  rate_down = 0.1;
}
```

3.5 Cell adhesion

Situation: cells need to adhere in order to be activated.

Solution: add a node that describes an adhesion state; possibly several nodes if there are several region of grouped adhesive cells, similar to compartments (see "Spatial organization" above).

4 COMPARISON BETWEEN FORMALISMS

The translation of biological knowledge into a mathematical model will take different forms according to the types of questions that the model is expected to address, the availability of knowledge about the molecular processes that the model is describing and the available data to compare the outputs with. The choice of the mathematical formalism is the very first step and should be decided based on the above-mentioned considerations.

In some cases, a formalism based on chemical kinetics might be required (especially when the expected results focus on drug dosage or timing of some events), and in other cases, a logical formalism might be enough to tackle the problem (e.g., when the model recapitulates the necessary cell conditions for a decision to proliferation or die). MaBoSS was initially built to fill the gap between ODEs and Boolean models, keeping the simplicity of logical models but allowing the introduction of real time into these models.

Hereafter, we illustrate , with two small examples of biological motifs, the challenges that one has to face when constructing models in ordinary differential equations and in logical formalism, and we further explain how the results of the stochastic approach can be interpreted.

In Figure S2, two common motifs, a positive feedback loop and a negative feedback loop, are compared.

The examples are based on interactions between proteins of the cell cycle and are largely inspired from models developed by John Tyson and Bela Novak (Novák and Tyson, 2008). The positive feedback loop involves two important components, Cyclin B, which, when in complex with its CDK partner, is fully active in M phase, and CDH1 (the human-homolog is FZR1), an auxiliary protein that mediates the degradation of the Cyclins. The second example shows a negative feedback loop, where Cdc20, another auxiliary protein that is indirectly activated by the Cyclins themselves through an intermediary enzyme. This intermediate is acting as a delay to allow the activation of the inhibitor, hence it is dispensable in the corresponding logical model.

In the ODE models, the parameters were chosen to obtain the desired phenotypes. For models of this size, it is doable but on models of higher dimension, it becomes more and more difficult.

4.1 Positive feedback loop

The ordinary differential equations of the positive feedback loop are the following:

```
CycB'= k1 - (k2p+k2*CDH1)*CycB
CDH1'= k3*(1-CDH1)/(Ja+1-CDH1) - k4*CycB*CDH1/(Ji+CDH1)
```

with the corresponding parameters that were chosen to show the two stable behaviors.

```
k1=0.15, k2p=0.01, k2=1.5, k3=0.1, k4=1, Ja=0.01, Ji=0.01
```

For the left panel, the initial conditions are set to an M-phase like condition with high Cyclin B and low CDH1 level, while for the right panel, the initial conditions are set to a G1-phase-like condition with a high level of CDH1 and a low level of Cyclin B. The initial conditions are determining in each state we are: the system remains in its initial state if no other external signal allows the jump from one stable state to another.

For the corresponding logical model, the logical rules are as follows:

```
CycB = !CDH1
CDH1 = !CycB
```

The Boolean rules are straightforward here and the solution space, referred to as the state transition graph, shows that when the system starts with an initial condition set to [CDH1 CycB] = [00], the system can either activate CDH1 or CycB, and with [CDH1 CycB] = [11], the system can either inactivate CDH1 or CycB, confirming the system bistability.

In MaBoSS, the model is written as follows:

```
Node CycB {
logic = (!CDC20 & !CDH1);
rate_up = @logic ? $u_CycB : 0;
rate_down = @logic ? 0 : $d_CycB;}

Node CDH1 {
logic = (!CycB);
rate_up = @logic ? $u_CDH1 : 0;
rate_down = @logic ? 0 : $d_CDH1;}
```

The transition rates `$u_CDH1,$d_CDH1,$u_CycB,`and `$u_CycB` are set to the default value 1. The two curves correspond to the probability for the state over time. They are superimposed: in red, prob[CycB] means that there are 50% chances that the state [CDH1 CycB] = [01] is reached and in green, prob[CDH1] means that there are 50% chances that the state [CDH1 CycB] = [10] is reached. Note that the probabilities of the stable states add to 1 in the absence of limit cycles.

4.2 Negative feedback loop

The ordinary differential equations of the negative feedback loop are the following:

```
dCYCB/dt= K1 - (k2a+K2*CDC20)*CycB
dCDC20/dt=k1S2*IEP*(1-CDC20)/(JaS2+1-CDC20) - k2S2*CDC20/(JiS2+CDC20)
dIEP/dt=k1IP*CycB - k2IP*IEP
```

with the corresponding parameters that were chosen to show the oscillatory behavior.

```
K1=0.15, K2a=0.01, K2=1.5
k1S2=1, k2S2=0.7, JaS2=0.01, JiS2=0.01
k1IP=1, k2IP=0.3
```

For the corresponding logical model, the logical rules are as follows:

```
CycB = !Cdc20
Cdc20 = CycB
```

The state transition graph shows the presence of a limit cycle attractor, where the same consecutive states are visited infinitely without any other way of escaping it.

In MaBoSS, the model is written as follows:

```
Node CycB {
logic = (!CDC20 & !CDH1);
rate_up = @logic ? $u_CycB : 0;
rate_down = @logic ? 0 : $d_CycB;}

Node CDC20 {
logic = (CycB);
rate_up = @logic ? $u_CDC20 : 0;
rate_down = @logic ? 0 : $d_CDC20;}
```

The transition rates are also set to the default value 1. The solution shows damped oscillations confirming the presence of a limit cycle.

REFERENCES

Novák, B. and Tyson, J. J. (2008). Design principles of biochemical oscillators. *Nature reviews Molecular cell biology* 9, 981–991

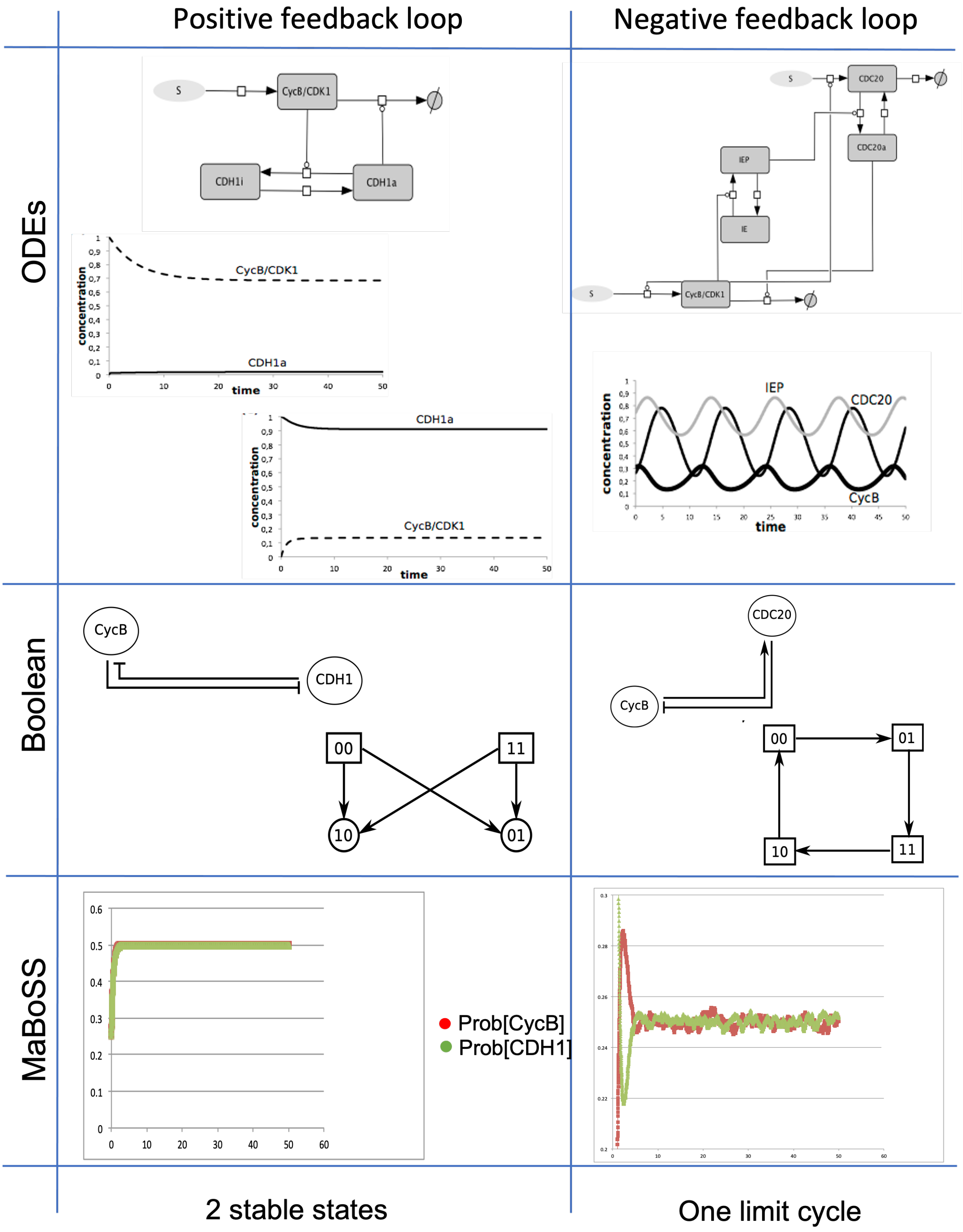

Figure S2. Simulations of the behaviors of simple positive and negative circuits, using three different formalisms: chemical kinetics (Ordinary differential equations), discrete (logical rules) and Boolean with stochastic approach (MaBoSS). In the upper panels, the reaction network and the simulations are shown, in the middle panels, the influence network and the state transition graphs are shown, and the lower panels, the probability trajectories of the stochastic simulations are shown.
